# Minichromosome Maintenance (MCM) Family as potential diagnostic and prognostic tumor markers for human gliomas

**DOI:** 10.1186/1471-2407-14-526

**Published:** 2014-07-21

**Authors:** Cong Hua, Gang Zhao, Yunqian Li, Li Bie

**Affiliations:** 1 Department of Neurosurgery of the First Clinical Hospital, Jilin University, 71 Xinmin St, Changchun, Jilin 130021, China; 2 Department of Pathology and Laboratory Medicine, University of California, Irvine, USA

**Keywords:** Glioma, MCMs, Tumor marker, Prognosis

## Abstract

**Background:**

Gliomas are the most common type of all central nervous system tumors. Almost all patients diagnosed with these tumors have a poor prognostic outcome. We aimed to identify novel glioma prognosis-associated candidate genes.

**Methods:**

We applied WebArrayDB software to span platform integrate and analyze the microarray datasets. We focused on a subset of the significantly up-regulated genes, the minichromosome maintenance (MCM) family. We used frozen glioma samples to predict the relationship between the expression of MCMs and patients outcome by qPCR and western blot.

**Results:**

We found that MCMs expression was significantly up-regulated in glioma samples. MCM2-7 and MCM10 expressions were associated with WHO tumor grade. High MCM2 mRNA expression appeared to be strongly associated with poor overall survival in patients with high grade glioma. Furthermore, we report that MCM7 is strongly correlated with patient outcome in patients with WHO grade II-IV tumor. MCM3 expression was found to be up-regulated in glioma and correlated with overall survival in patients with WHO grade III tumor. MCM2, MCM3 and MCM7 expression levels were of greater prognostic relevance than histological diagnosis according to the current WHO classification system.

**Conclusions:**

High expression of MCM 2, MCM3 and MCM7 mRNA correlated with poor outcome and may be clinically useful molecular prognostic markers in glioma.

## Background

Gliomas are one of the most common malignant tumors of the central nervous system. Gliomas represent a group of low grade and high grade brain tumors that originate from glial cells. Most are characterized by diffuse infiltrative growth in the surrounding brain. According to the 2007 WHO grading system [1], these tumors are classified as typical WHO grade I-IV. The median survival for patients with anaplastic astrocytoma (WHO grade III) and glioblastoma (WHO grade IV) is less than threes years and less than one year, respectively [2]. The prognosis remains poor despite extensive research and advances in radiation therapy and chemotherapeutic regimes [3]. All types of cancer as well as glioma constitute a major public health problem that presents several challenges to researchers such as identification of biomarkers for improved and early diagnosis, classification of tumors, and the definition of targets for more effective treatment. Genetic analysis over the past 30 years has defined the major mutational targets in the human genome that are associated with the formation of glioma [4]. Moreover, large-scale association genome-wide surveys have been used to identify new biomarkers that have been developed as diagnostic and prognostic tools.

In this study, to unravel molecular glioma tumorigenesis and discover novel molecular biomarkers for diagnostic and/or prognostic purposes, we apply WebArrayDB software (http://www.WebArrayDB.org) to span platform integrate and analyze the microarray datasets [5-7], including data stored at the Cancer Genome Anatomy Project (http://cgap.nci.nih.gov/Genes).

Among the genes up-regulated in gliomas, we focused on a subset of the significantly up-regulated genes, the minichromosome maintenance (MCM) family. The MCM family includes eight members: MCM2-MCM7 and MCM10 [8]. They are considered to function as licensing components for the S-phase of cell cycle [9]. MCMs can be expressed in abundance in different cell cycles and degraded in quiescence, senescence and differentiation steps thus they can be used as specific markers of the cell cycle state in tissues [10]. This feature of MCM genes in proliferating cells has led to their potential clinical application as a marker for cancer screening [11].

Here we report that MCM family genes are almost up-regulated in 59 human glioma tumor samples compared to six normal brain controls by qRT-PCR. Our study indicates for the first time that high MCM2 mRNA expression appears to be strongly associated with poor overall survival in high grade glioma. Furthermore, we report that MCM7 is strongly correlated with patient outcome in WHO grade II-IV. MCM3 expression found to be up-regulated in glioma and correlated with overall survival in WHO grade III. MCM2, MCM3 and MCM7 expression level were of greater prognostic relevance than histological diagnosis according to the current WHO classification system. Interestingly, in the last few years, studies have pointed out the roles of MCM family members as diagnostic and prognostic markers for several malignancies [12].

## Methods

### Patients and tumor samples

A total of 59 glioma specimens were obtained from the Department of Neurosurgery, First Affiliated Hospital of Jilin University, from 2003 to 2012. The samples included 21 females and 38 males and an age range 2–69 years. Samples were collected immediately after surgical resection, snap frozen, and stored at -80°C until used for RNA extraction. All gliomas were samples of primary tumor before therapy. All cases had been diagnosed at the primary hospital by neuropathologists. Original pathology slides were obtained and reviewed blinded to the original diagnosis according to the 2007 WHO classification (14 II grade, 21 III grade and 24 IV grade). The samples consisted of astrocytoma (n = 27), anaplastic astrocytoma (n = 9) and glioblastoma (n = 23). Six control samples (normal brain) were obtained from the Department of Neurosurgery, First Affiliated Hospital of Jilin University from patients undergoing surgery for brain trauma (n = 4) and epilepsy (n = 2). They were all reviewed to verify the absence of tumor. Written informed consent for participation in the study was obtained from all participants or their guardians. The study was approved by the Institutional Review Board (IRB) of The First Hospital of Jilin University (IRB00008484).

### RNA isolation and quality evaluation

Total RNA was extracted from frozen sections with the TRIzol Reagent (Invitrogen, Carlsbad, CA, USA) following the manufacturer’s protocol. Total RNA yield was measured with an A260/280 ratio of 1.7-1.9, demonstrating purity. Quality was evaluated on nanochips with the Bio-Rad Experion automated electrophoresis system (Bio-Rad Laboratories, Hercules, CA, USA). Samples had a 28S/18S ratio of 1.5 and did not show evidence of ribosomal peak degradation.

### Real-time quantitative reverse transcription PCR

About 500 ng total RNA from each sample was reverse transcribed with SuperScript II Reverse Transcriptase (Invitrogen, Carlsbad, CA, USA). Primers were designed with AlleleID Version 7.0 software (Premierbiosoft, Palo Alto, CA, USA). Predesigned gene expression assays were obtained from Integrated Device Technology (IDT, San Diego, CA, USA). Real-time quantitative PCR was then carried out using an ABI Prism 7900 Sequence Detection System (Applied Biosystems, Carlsbad, CA, USA). A quantity of 15 ng of cDNA was used in a 25 μl PCR reaction containing the appropriate primers and 1 × SYBR Green PCR Super mix (BioPioneer, San Diego, CA, USA). All products were 77 to 134 bp. Dissociation curves of each sample were used to check the specificity of amplification. PCR reactions were examined by 1.6% agarose gel electrophoresis for verification of dissociation curve results. Parallel experiments were carried out using an 18S rRNA and HPRT1 primer set. qPCR reactions were performed in triplicate, and the relative fold changes were calculated with the Pfaffl method [13] for each gene corrected using 18S rRNA + HPRT1[14]. All primer pairs utilized in this study presented amplification efficiency between 91-110% (Additional file 1).

### Western blot

To detect MCM2, MCM3 and MCM7 protein in glioma tumors, we examined six glioma samples (two each of WHO stages II, III, IV). The protein concentration of cell lysates was determined by the Bio-Rad Protein Assay (Bio-Rad Laboratories, Hercules, CA, USA) according to the manufacturer’s instructions. Thirty micrograms of protein per tissue lysate was electrophoresed on 7-12% gels and transferred to a polyvinylidene difluoride membrane filter (Millipore, Billerica, MA, USA). Blots were probed with the appropriate antibody. Quantitative determination of the protein was assessed by densitometric scanning of the band from film. An AlphaImager 2000 Documentation and Analysis System (Alpha Innotech Corp, San Leandro, CA, USA) was used to quantify bands of appropriate sizes.

The following primary antibodies were used: anti-*β*-actin (Sigma Chemical Co, St. Louis MO, USA), anti-MCM2 (sc-10771, Santa Cruz Biotechnology, USA), anti-MCM3 (sc-9849, Santa Cruz Biotechnology, USA), and anti-MCM7 (sc-22782, Santa Cruz Biotechnology, USA).

### Statistical analysis

The statistical significance of differences in the means was calculated using the ANVOA test. To identify a model describing the relationship between survival and the MCMs mRNA expression, the functional form of the relationship was tested by maximally selected log-rank statistics as previously described [15]. The resulting model was applied in further survival analyses. Multivariate Cox regression was used to investigate the prognostic power of candidate gene expression adjusting for established prognostic variables. The Cox proportional hazards regression were carried out with the use of the design and survival package of the R software environment. Overall survival curves (from diagnosis to death) were obtained using the Kaplan-Meier method and compared with a log-rank test. A *p* value of less than 0.05 was considered to indicate statistical significance.

## Results

### Up-regulation of the MCM family expression in human glioma samples

**Figure 1:**
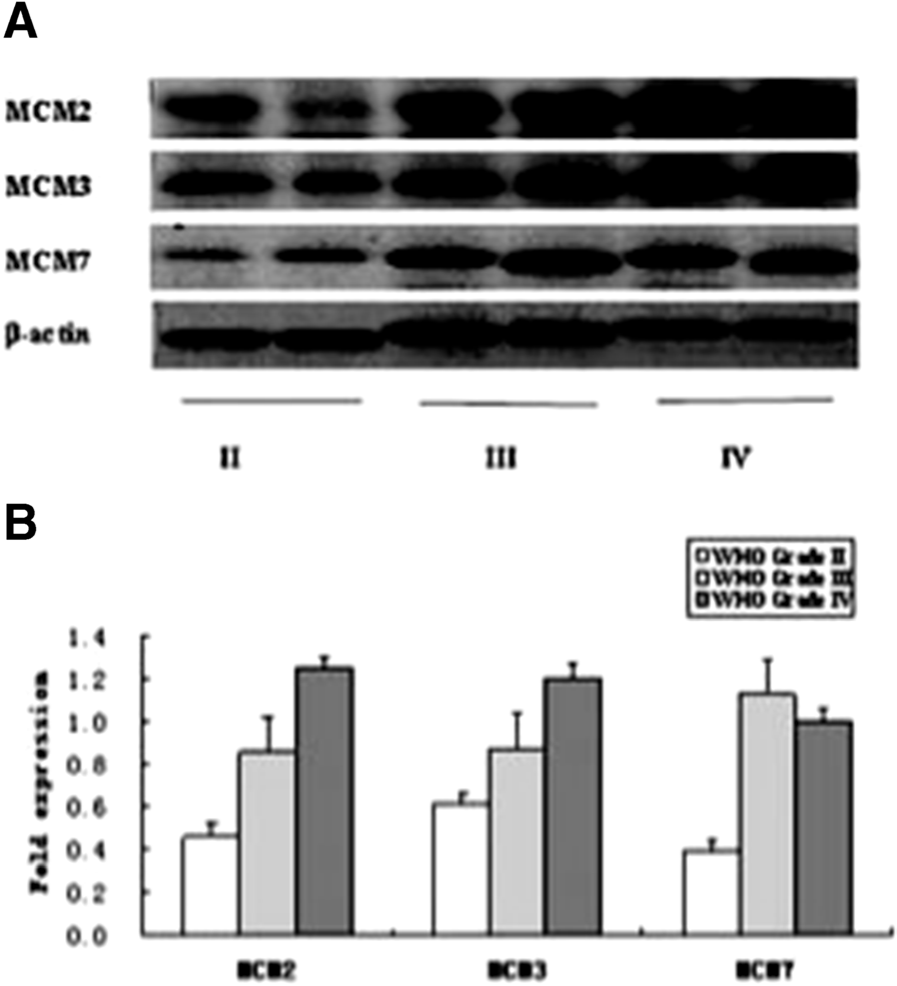
**Expression of MCM2, MCM3 and MCM7 protein in gliomas by Western blot. A** western blots of six glioma samples (WHO grade II, III and IV). **B** Quantitation of the western blots by using *β*-actin as the loading control.

The mRNA expression level of the MCM family members were examined in 59 human glioma samples and six normal brain tissues by qPCR (n = 3), normalized to 18S rRNA and HPRT1 levels. We found a significant increase in MCM2 (3.5 fold), MCM3 (3.1 fold), MCM4 (4.3 fold), MCM5 (3.8 fold), MCM6 (3.5 fold), MCM7 (3.0 fold) and MCM10 (3.6 fold) expressions in tumor tissues (the average fold value of three grades) compared to the normal brain controls. Moreover, as shown in Table 1, we observed that MCM3-MCM5,MCM7 and MCM10 were significantly changed between grade II, III and IV samples (*p* < 0.05). MCM2 and MCM6 were significantly increased between grade II (low grade) and III-IV (high grade) samples (*p* < 0.05). Four independent external microarray datasets (8 normal, 29 stage II, 116 stage III and 618 stage IV, Additional file 2) were analyzed using the WebArrayDB cross-platform analysis suite to validate our experiment results. Genes were sorted in ascending order according to the *p* values for WHO grade, after taking into account gender and patient age as variables. Expression of MCM family members was highly correlated with glioma grade (Table 1). To confirm the specificity of the qPCR results, we characterized MCM2, MCM3, MCM7, as well as *β*-actin as a loading control, in six glioma samples for which freshly frozen materials were available. As shown in Figure 1, an immunoreactive band of MCM2, MCM3 and MCM7 were seen in all six cases of gliomas, MCM2, MCM3 and MCM7 expressions were significantly higher in malignant tissues than in tissues with low malignant potential.

**Table 1:** Comparison of mRNA expression of MCMs in glioma tumor tissue by qRT-PCR and microarray analysis

**Gene**	**Unique ID**	**Gene origin**	**Fold**							***p *** **value**					
			**II/Normal**	**III/Normal**	**IV/Normal**	**III/II**	**IV/II**	**IV/III**	**(II-IV)/Normal**	**II → Normal**	**III → Normal**	**IV → Normal**	**III → II**	**IV → II**	**IV → III**
MCM2	202107_s_at	qRT-PCR	1.91	3.9	4.22	2.04	2.21	1.08	3.5	1.27E-02	1.38E-04	5.43E-10	1.20E-04	4.91E-10	3.84E-01
		Microarray	1.61	3.68	5.39	2.16	3.16	1.47		2.84E-02	5.22E-05	8.61E-09	5.33E-09	0.00E + 00	1.53E-05
MCM3	201555_at	qRT-PCR	1.59	3.01	4.46	1.89	2.81	1.49	3.1	1.08E-01	2.51E-02	8.25E-10	2.00E-02	1.46E-11	3.86E-03
		Microarray	1.92	2.32	2.79	1.26	1.52	1.2		1.57E-02	1.14E-04	2.45E-07	3.24E-02	1.18E-07	3.26E-03
MCM4	222036_s_at	qRT-PCR	2.41	4.56	6.15	1.89	2.55	1.35	4.3	1.14E-03	2.42E-04	2.11E-06	6.07E-04	2.33E-07	2.81E-02
		Microarray	0.81	1.51	1.61	1.58	1.69	1.07		1.00E + 00	5.07E-01	2.01E-01	9.64E-06	6.36E-09	7.04E-01
MCM5	201755_at	qRT-PCR	1.91	4.25	4.48	2.22	2.34	1.05	3.8	1.49E-02	2.38E-04	5.92E-07	3.32E-05	1.49E-02	1.43E-02
		Microarray	0.57	1.95	2.46	2.03	2.57	1.26		7.88E-01	5.97E-01	1.14E-01	1.19E-04	7.13E-10	1.23E-01
MCM6	201930_at	qRT-PCR	1.28	4.8	4.63	3.76	3.62	0.96	3.5	4.46E-01	4.46E-05	8.26E-09	2.65E-07	6.85E-12	7.04E-01
		Microarray	2.24	2.63	3.05	2.07	2.4	1.16		2.82E-03	1.83E-02	3.22E-02	4.30E-13	0.00E + 00	7.39E-02
MCM7	208795_s_at	qRT-PCR	1.51	3.29	4.33	2.17	2.86	1.32	3.0	3.39E-01	1.82E-02	1.34E-08	1.25E-02	5.59E-09	4.95E-02
		Microarray	1.02	2.23	2.08	1.79	1.67	0.93		1.00E + 00	8.94E-04	1.93E-03	5.57E-10	6.93E-10	6.36E-01
MCM10	220651_s_at	qRT-PCR	2.58	3.86	4.73	1.5	1.83	1.22	3.6	5.72E-05	3.31E-04	3.20E-08	8.96E-03	4.41E-07	6.48E-02
		Microarray	6.18	26.21	31.81	3.54	4.29	1.21		1.46E-02	6.54E-09	3.05E-10	4.62E-07	1.77E-11	5.83E-01

MCM3 and MCM5,MCM7 and MCM10 were significant different between grade II, III and IV samples (*p* < 0.05). MCM2 and MCM6 expressions were significantly increased between grade II (low grade) and III-IV (high grade) samples (*p* < 0.05).

### Multivariate survival analysis of prognostic parameters

Multivariate survival analysis identified high expression of MCM2 as an independent prognostic factor for survival time, as well as MCM3 and MCM7 (*p* < 0.05), (Table 2).

**Table 2:** Multivariate analysis on prognosis of the patients (n = 59)

**Parameter**	**Risk ratio**	**95% CI**	***p***
Sex	2.067	0.789-4.670	0.151
Age (≥40)	3.155	1.984-4.039	0.042
WHO (Grade 4)	3.0843	1.523-3.810	0.034
MCM2	8.519	6.280-10.508	0.004
MCM3	7.328	5.102-9.833	0.007
MCM4	0.506	0.843-1.441	0.477
MCM5	2.117	0.946-3.127	0.146
MCM6	2.873	1.377-4.073	0.090
MCM7	6.576	4.114-8.243	0.010
MCM10	0.029	0.015-1.093	0.937

### The MCMs expression correlates with poor outcome in patients with glioma

**Figure 2:**
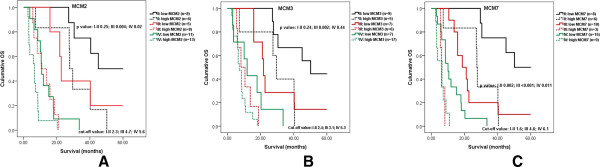
**Kaplan-Meier estimates of overall survival (OS) in relation to MCM2, MCM3 and MCM7 expression as determined by qPCR.** Cutoff value for dichotomization of MCM2, MCM3 and MCM7 expression and *p* values were determined by maximally selected log-rank statistics. **A** High MCM2 expression appeared to be strongly associated with poor overall survival in high grade glioma. **B** MCM3 expression was to be up-regulated in glioma and correlated with OS in the grade III group, In the grade III group, the expression of three genes was significantly associated with patient outcome (*p* < 0.01); **C** MCM7 expression appeared to be a strongly positive prognostic factor in the WHO grade II-IV group (*p* < 0.01).

We reviewed each grade of tumor separately and investigated whether the expression of MCM2, MCM3 and MCM7 predicts patient survival within each subgroup. The search for a model describing the relationship between survival and expression of MCM family genes expression using maximally selected log-rank statistics identified a cutoff model to be most suitable: MCM2 (fold change cutoff for stage II 2.3, III 4.7, and IV 5.6,), MCM3(II 2.4, III 3.1, and IV 5.3) and MCM7 (II 1.6, III 4.6, and IV 6.1). The qPCR cutoff ratios were used to separate two patient subgroups with significantly different outcomes [maximally selected log rank statistics, *p* < 0.05 (overall survival)]. Applying these MCM family expression cutoff to Kaplan-Meier survival curve estimation revealed decreased survival probability for patients with tumor expressing high levels of MCM2, MCM3 and MCM7 (*p* < 0.05), (Figure 2). In the grade II group, MCM7 expression appeared to be a strongly positive prognostic factor (*p* < 0.01). In the grade III group, better prognosis was found for patients with glioma with low expression of three genes compared to patients with high expression (*p* < 0.01). MCM2 and MCM7 expression appeared to be prognostic factors in the grade IV group, although they are not strongly positive in any of these cases (*p* < 0.05). Thus the expression level of MCM2, MCM3 and MCM7 reveal information on the survival probability for patients with glioma beyond that revealed by grade alone.

The result was validated by GBM database. Repository of Molecular Brain Neoplasia Data (Rembrandt, https://caintegrator.nci.nih.gov/rembrandt/), a cancer clinical genomics database and a Web-based data mining and analysis platform aimed at facilitating discovery by connecting the dots between clinical information and genomic characterization data. The microarray data are available for 181 GBM samples in Rembrandt database. MCM2 and MCM3 were not correlate with the prognosis of GBM patients (*p* > 0.05). When the cut-off point was the up-regulation of 2 fold, MCM7 was significant relative to the outcome of GBM patients (*p* < 0.01), (Table 3).

**Table 3:** **Comparison of mRNA expression of MCM2, MCM3 and MCM7 in glioma tumor tissue by microarray analysis (Rembrandt,**https://caintegrator.nci.nih.gov/rembrandt/**)**

**Gene**	**Unique ID**	**Gene origin**	**Number of samples (all upregulate)**		**Fold**	***p *** **value**
			**Group 1**	**Group 2**	**Cut-off point**	
MCM2	202107_s_at	Microarray	133	48	2.0	0.181
MCM3	201555_at	Microarray	138	43	2.0	0.417
MCM7	208795_s_at	Microarray	59	122	3.0	0.009

*p* < 0.05.

MCM2 and MCM3 were not correlate with the prognosis of GBM patients (*p* > 0.05). When the cutoff point was the up-regulation of 3fold, MCM7 was significant correlate with the outcome of GBM patients (*p* < 0.01).

## Discussion

Glioma tumors are the most common and deadly brain tumors. However, the survival of patients with low grade (stage II) glioma is significantly higher than that of patients with high grade (stage III-IV) tumors. In the study, we applied WebArrayDB software (http://www.WebArrayDB.org) to analysis three independent microarray datasets which come from different microarray platforms. We focused on the part of the members of the MCM gene family most highly significantly up-regulated in gliomas.

MCMs were first discovered in the mutants of the yeast Saccharomyces cerevisiae that had defects in maintaining a simple minichromosome [16]. MCM 2–7 form a heterohexamer complex [17]. The MCM protein complex is associated with the origins of DNA replication to form part of the pre-replicative complex. Activation of MCM complex by cyclin-dependent kinases, such as Cdc6, Cdt1 andDbf4/Cdc7, leads to initiation of DNA synthesis [18]. The hexameric MCM component of the pre-replicative complex exhibits helicase activity that may make DNA unwind during replication [11]. Thus, MCM proteins allow the DNA replication machinery to access binding sites on DNA [19]. Several studies suggested that increased levels of MCMs can identify not only malignant cells [20-25] but also precancerous cells and tumor recurrence [26-28], indicating that they might also serve as prognostic tumor markers. The last member, MCM10, may have a dual function: firstly to stabilize DNA polymerase-α-primase and secondly to target it to chromatin [29].

Recent studies have proven that MCMs expression has a relationship with diagnosis and prognosis. MCM2 and MCM3 seem to have the most important role in several types of neoplasia, including alimentary system tumors [30-35], genitourinary system tumors [36-41], lung cancer [42,43], breast cancer [44-47], and meningioma [27,48]. Moreover, several investigators have found that the measurement of MCM6 protein expression is a good diagnostic marker for chondrosarcoma [49], and lymphoma [50]. MCM2, MCM3 and MCM7 proteins expression is also known to be an important tool for estimating tumor proliferation and a useful adjunct to the routinely used proliferation markers for glioma diagnosis [51-56].

Moreover, in past research, MCMs have also provided useful information on outcome of patients with glioma. Wharton *et al*. found that cases with a high MCM2 labeling index had a poorer prognosis than those with a low index in patients with oligodendroglioma [51]. Scott *et al*. reported that the cyclin A:MCM2 labeling fraction might predict a relatively favorable response to radical radiotherapy in patients with glioblastoma [52]. Söling *et al*. in their series of patients with astrocytoma found that high MCM3 expression is an independent predictor of poor outcome [53].

Compared with previous reports, we confirmed MCM 2, MCM 3 and MCM7 expression were correlated with WHO grading of glioma tumors on mRNA profiles by microarray data analysis and qPCR. The result of western blot also validated this finding. Some studies reported positive correlations in the expression of MCMs with Ki67 [44,47,57]. Moreover, several studies have revealed that MCMs are characterized by higher specificity and sensitivity than other conventional proliferative markers [58,59]. Ki-67 is a crucial molecular marker for estimating the progression and prognosis of gliomas [60,61]. Despite the fact that MCMs and Ki67 have a similar expression pattern during many cell cycle phases (G_1_, S, G_2_ and M phases), detailed cell-cycle analysis shows differences between both markers. Ki67 is absent during the early G_1_ phase, whereas MCMs are expressed in the entire G_1_ phase. As such, the degree of tumor cell proliferation may be better reflected by using MCMs.

Kaplan-Meier analysis showed that MCM2, MCM3 and MCM7 can function as an independent prognostic indicator for overall survival (*p* < 0.05). Our study indicates for the first time that high MCM2 mRNA expression appears to be strongly associated with poor overall survival in high grade glioma. MCM3 expression was found to be up-regulated in glioma and correlated with overall survival in the grade III group. However, MCM3 does not appear to be a predictor of survival in the patients of the grade IV group. Söling *et al*. have also reported results supporting this conclusion [53]. Likewise, MCM2 and MCM3 expression in grade II group does not appear to be a significant predictor, although this might be explained by the limited sample size. Furthermore, we report that MCM7 is correlated with patient outcome in grade II-IV group. Erkan EP *et al.* also report MCM7 was the significant up-regulate gene in GBM samples compared with normal white matter tissues. Moreover, siRNA-mediated knockdown of MCM7 expression reduced GBM cell proliferation and also inhibited tumor growth in both xenograte and orthotopic mouse models of GBM [56].

## Conclusions

In conclusion, the results suggest that MCM2, MCM3 and MCM7 play important roles in glioma tumor progression. Most notably, MCM2, MCM3 and MCM7 aberrations in glioma tumors portend a particularly aggressive clinical behavior.

## Abbreviations

MCM: Minichromosome maintenance; GBM: Glioblastoma; qRT-PCR: Quantitative real-time reverse transcription polymerase chain reaction assays; TCGA: The Cancer Genome Anatomy; Rembrandt: Repository of Molecular Brain Neoplasia Data; OS: Overall survival.

## Competing interests

The authors have no conflict of interest to declare.

## Authors’ contributions

CH, GZ and YQL carried out the molecular genetic studies, and participated in the preparation of the manuscript. LB conceived the study, and participated in its design and coordination and drafted the manuscript. All authors read and approved the final manuscript.

## Pre-publication history

The pre-publication history for this paper can be accessed here:

http://www.biomedcentral.com/1471-2407/14/526/prepub

## Supplementary Material

Additional file 1 Primer sequences and amplification summary. Click here for file

Additional file 2 The other most up-regulated 20 genes. Click here for file

## References

[B1] Louis DN Ohgaki H Wiestler OD Cavenee WK Burger PC Jouvet A Scheithauer BW Kleihues P The 2007 WHO classification of tumours of the central nervous system Acta Neuropathol 2007 114 2 97 109 17618441 10.1007/s00401-007-0243-4 PMC1929165

[B2] DeAngelis LM Brain tumors N Engl J Med 2001 344 2 114 123 11150363 10.1056/NEJM200101113440207

[B3] Jemal A Murray T Ward E Samuels A Tiwari RC Ghafoor A Feuer EJ Thun MJ Cancer statistics, 2005 CA Cancer J Clin 2005 55 1 10 30 15661684 10.3322/canjclin.55.1.10

[B4] Van Meir EG Hadjipanayis CG Norden AD Shu HK Wen PY Olson JJ Exciting new advances in neuro-oncology: the avenue to a cure for malignant glioma CA Cancer J Clin 2010 60 3 166 193 20445000 10.3322/caac.20069 PMC2888474

[B5] Freije WA Castro-Vargas FE Fang Z Horvath S Cloughesy T Liau LM Mischel PS Nelson SF Gene expression profiling of gliomas strongly predicts survival Cancer Res 2004 64 18 6503 6510 15374961 10.1158/0008-5472.CAN-04-0452

[B6] Gravendeel LA Kouwenhoven MC Gevaert O de Rooi JJ Stubbs AP Duijm JE Daemen A Bleeker FE Bralten LB Kloosterhof NK De Moor B Eilers PH van der Spek PJ Kros JM Sillevis Smitt PA van den Bent MJ French PJ Intrinsic gene expression profiles of gliomas are a better predictor of survival than histology Cancer Res 2009 69 23 9065 9072 19920198 10.1158/0008-5472.CAN-09-2307

[B7] Wong KK Chang YM Tsang YT Perlaky L Su J Adesina A Armstrong DL Bhattacharjee M Dauser R Blaney SM Chintagumpala M Lau CC Expression analysis of juvenile pilocytic astrocytomas by oligonucleotide microarray reveals two potential subgroups Cancer Res 2005 65 1 76 84 15665281

[B8] Johnson EM Kinoshita Y Daniel DC A new member of the MCM protein family encoded by the human MCM8 gene, located contrapodal to GCD10 at chromosome band 20p12.3-13 Nucleic Acids Res 2003 31 11 2915 2925 12771218 10.1093/nar/gkg395 PMC156728

[B9] Kearsey SE Maiorano D Holmes EC Todorov IT The role of MCM proteins in the cell cycle control of genome duplication Bioessays 1996 18 3 183 190 8867732 10.1002/bies.950180305

[B10] Stoeber K Tlsty TD Happerfield L Thomas GA Romanov S Bobrow L Williams ED Williams GH DNA replication licensing and human cell proliferation J Cell Sci 2001 114 Pt 11 2027 2041 11493639 10.1242/jcs.114.11.2027

[B11] Tachibana KE Gonzalez MA Coleman N Cell-cycle-dependent regulation of DNA replication and its relevance to cancer pathology J Pathol 2005 205 2 123 129 15643673 10.1002/path.1708

[B12] Giaginis C Vgenopoulou S Vielh P Theocharis S MCM proteins as diagnostic and prognostic tumor markers in the clinical setting Histol Histopathol 2010 25 3 351 370 20054807 10.14670/HH-25.351

[B13] Pfaffl MW A new mathematical model for relative quantification in real-time RT-PCR Nucleic Acids Res 2001 29 9 e45 11328886 10.1093/nar/29.9.e45 PMC55695

[B14] Valente V Teixeira SA Neder L Okamoto OK Oba-Shinjo SM Marie SK Scrideli CA Paco-Larson ML Carlotti CG Jr Selection of suitable housekeeping genes for expression analysis in glioblastoma using quantitative RT-PCR BMC Mol Biol 2009 10 17 19257903 10.1186/1471-2199-10-17 PMC2661085

[B15] Hothorn T Zeileis A Generalized maximally selected statistics Biometrics 2008 64 4 1263 1269 18325074 10.1111/j.1541-0420.2008.00995.x

[B16] Tye BK MCM proteins in DNA replication Annu Rev Biochem 1999 68 649 686 10872463 10.1146/annurev.biochem.68.1.649

[B17] Bell SP Dutta A DNA replication in eukaryotic cells Annu Rev Biochem 2002 71 333 374 12045100 10.1146/annurev.biochem.71.110601.135425

[B18] Maiorano D Lutzmann M Mechali M MCM proteins and DNA replication Curr Opin Cell Biol 2006 18 2 130 136 16495042 10.1016/j.ceb.2006.02.006

[B19] Laskey RA Madine MA A rotary pumping model for helicase function of MCM proteins at a distance from replication forks EMBO Rep 2003 4 1 26 30 12524516 10.1038/sj.embor.embor706 PMC1315806

[B20] Freeman A Morris LS Mills AD Stoeber K Laskey RA Williams GH Coleman N Minichromosome maintenance proteins as biological markers of dysplasia and malignancy Clin Cancer Res 1999 5 8 2121 2132 10473096

[B21] Going JJ Keith WN Neilson L Stoeber K Stuart RC Williams GH Aberrant expression of minichromosome maintenance proteins 2 and 5, and Ki-67 in dysplastic squamous oesophageal epithelium and Barrett’s mucosa Gut 2002 50 3 373 377 11839717 10.1136/gut.50.3.373 PMC1773132

[B22] Ishimi Y Okayasu I Kato C Kwon HJ Kimura H Yamada K Song SY Enhanced expression of Mcm proteins in cancer cells derived from uterine cervix Eur J Biochem 2003 270 6 1089 1101 12631269 10.1046/j.1432-1033.2003.03440.x

[B23] Meng MV Grossfeld GD Williams GH Dilworth S Stoeber K Mulley TW Weinberg V Carroll PR Tlsty TD Minichromosome maintenance protein 2 expression in prostate: characterization and association with outcome after therapy for cancer Clin Cancer Res 2001 7 9 2712 2718 11555583

[B24] Ramnath N Hernandez FJ Tan DF Huberman JA Natarajan N Beck AF Hyland A Todorov IT Brooks JS Bepler G MCM2 is an independent predictor of survival in patients with non-small-cell lung cancer J Clin Oncol 2001 19 22 4259 4266 11709570 10.1200/JCO.2001.19.22.4259

[B25] Rodins K Cheale M Coleman N Fox SB Minichromosome maintenance protein 2 expression in normal kidney and renal cell carcinomas: relationship to tumor dormancy and potential clinical utility Clin Cancer Res 2002 8 4 1075 1081 11948116

[B26] Alison MR Hunt T Forbes SJ Minichromosome maintenance (MCM) proteins may be pre-cancer markers Gut 2002 50 3 290 291 11839701 10.1136/gut.50.3.290 PMC1773123

[B27] Hunt DP Freeman A Morris LS Burnet NG Bird K Davies TW Laskey RA Coleman N Early recurrence of benign meningioma correlates with expression of mini-chromosome maintenance-2 protein Br J Neurosurg 2002 16 1 10 15 11928726 10.1080/02688690120114174

[B28] Tan DF Huberman JA Hyland A Loewen GM Brooks JS Beck AF Todorov IT Bepler G MCM2–a promising marker for premalignant lesions of the lung: a cohort study BMC Cancer 2001 1 6 11472637 10.1186/1471-2407-1-6 PMC35283

[B29] Ricke RM Bielinsky AK Mcm10 regulates the stability and chromatin association of DNA polymerase-alpha Mol Cell 2004 16 2 173 185 15494305 10.1016/j.molcel.2004.09.017

[B30] Tokuyasu N Shomori K Nishihara K Kawaguchi H Fujioka S Yamaga K Ikeguchi M Ito H Minichromosome maintenance 2 (MCM2) immunoreactivity in stage III human gastric carcinoma: clinicopathological significance Gastric Cancer 2008 11 1 37 46 18373176 10.1007/s10120-008-0451-1

[B31] Scott IS Morris LS Bird K Davies RJ Vowler SL Rushbrook SM Marshall AE Laskey RA Miller R Arends MJ Coleman N A novel immunohistochemical method to estimate cell-cycle phase distribution in archival tissue: implications for the prediction of outcome in colorectal cancer J Pathol 2003 201 2 187 197 14517835 10.1002/path.1444

[B32] Guzinska-Ustymowicz K Stepien E Kemona A MCM-2, Ki-67 and PCNA protein expressions in pT3G2 colorectal cancer indicated lymph node involvement Anticancer Res 2008 28 1B 451 457 18383884

[B33] Giaginis C Georgiadou M Dimakopoulou K Tsourouflis G Gatzidou E Kouraklis G Theocharis S Clinical significance of MCM-2 and MCM-5 expression in colon cancer: association with clinicopathological parameters and tumor proliferative capacity Dig Dis Sci 2009 54 2 282 291 18465232 10.1007/s10620-008-0305-z

[B34] Freeman A Hamid S Morris L Vowler S Rushbrook S Wight DG Coleman N Alexander GJ Improved detection of hepatocyte proliferation using antibody to the pre-replication complex: an association with hepatic fibrosis and viral replication in chronic hepatitis C virus infection J Viral Hepat 2003 10 5 345 350 12969185 10.1046/j.1365-2893.2003.00454.x

[B35] Marshall A Rushbrook S Morris LS Scott IS Vowler SL Davies SE Coleman N Alexander G Hepatocyte expression of minichromosome maintenance protein-2 predicts fibrosis progression after transplantation for chronic hepatitis C virus: a pilot study Liver Transpl 2005 11 4 427 433 15776414 10.1002/lt.20347

[B36] Gakiopoulou H Korkolopoulou P Levidou G Thymara I Saetta A Piperi C Givalos N Vassilopoulos I Ventouri K Tsenga A Bamias A Dimopoulos MA Agapitos E Patsouris E Minichromosome maintenance proteins 2 and 5 in non-benign epithelial ovarian tumours: relationship with cell cycle regulators and prognostic implications Br J Cancer 2007 97 8 1124 1134 17940502 10.1038/sj.bjc.6603992 PMC2360432

[B37] Scott IS Heath TM Morris LS Rushbrook SM Bird K Vowler SL Arends MJ Coleman N A novel immunohistochemical method for estimating cell cycle phase distribution in ovarian serous neoplasms: implications for the histopathological assessment of paraffin-embedded specimens Br J Cancer 2004 90 8 1583 1590 15083189 10.1038/sj.bjc.6601660 PMC2409706

[B38] Kato K Toki T Shimizu M Shiozawa T Fujii S Nikaido T Konishi I Expression of replication-licensing factors MCM2 and MCM3 in normal, hyperplastic, and carcinomatous endometrium: correlation with expression of Ki-67 and estrogen and progesterone receptors Int J Gynecol Pathol 2003 22 4 334 340 14501812 10.1097/01.pgp.0000092129.10100.5e

[B39] Dudderidge TJ McCracken SR Loddo M Fanshawe TR Kelly JD Neal DE Leung HY Williams GH Stoeber K Mitogenic growth signalling, DNA replication licensing, and survival are linked in prostate cancer Br J Cancer 2007 96 9 1384 1393 17406359 10.1038/sj.bjc.6603718 PMC2360172

[B40] Burger M Denzinger S Hartmann A Wieland WF Stoehr R Obermann EC Mcm2 predicts recurrence hazard in stage Ta/T1 bladder cancer more accurately than CK20, Ki67 and histological grade Br J Cancer 2007 96 11 1711 1715 17505513 10.1038/sj.bjc.6603784 PMC2359908

[B41] Kruger S Thorns C Stocker W Muller-Kunert E Bohle A Feller AC Prognostic value of MCM2 immunoreactivity in stage T1 transitional cell carcinoma of the bladder Eur Urol 2003 43 2 138 145 12565771 10.1016/s0302-2838(02)00580-8

[B42] Yang J Ramnath N Moysich KB Asch HL Swede H Alrawi SJ Huberman J Geradts J Brooks JS Tan D Prognostic significance of MCM2, Ki-67 and gelsolin in non-small cell lung cancer BMC Cancer 2006 6 203 16882345 10.1186/1471-2407-6-203 PMC1555597

[B43] Hashimoto K Araki K Osaki M Nakamura H Tomita K Shimizu E Ito H MCM2 and Ki-67 expression in human lung adenocarcinoma: prognostic implications Pathobiology 2004 71 4 193 200 15263808 10.1159/000078673

[B44] Wojnar A Pula B Piotrowska A Jethon A Kujawa K Kobierzycki C Rys J Podhorska-Okolow M Dziegiel P Correlation of intensity of MT-I/II expression with Ki-67 and MCM-2 proteins in invasive ductal breast carcinoma Anticancer Res 2011 31 9 3027 3033 21868554

[B45] Gonzalez MA Pinder SE Callagy G Vowler SL Morris LS Bird K Bell JA Laskey RA Coleman N Minichromosome maintenance protein 2 is a strong independent prognostic marker in breast cancer J Clin Oncol 2003 21 23 4306 4313 14645419 10.1200/JCO.2003.04.121

[B46] Shetty A Loddo M Fanshawe T Prevost AT Sainsbury R Williams GH Stoeber K DNA replication licensing and cell cycle kinetics of normal and neoplastic breast Br J Cancer 2005 93 11 1295 1300 16278669 10.1038/sj.bjc.6602829 PMC2361513

[B47] Werynska B Pula B Muszczynska-Bernhard B Piotrowska A Jethon A Podhorska-Okolow M Dziegiel P Jankowska R Correlation between expression of metallothionein and expression of Ki-67 and MCM-2 proliferation markers in non-small cell lung cancer Anticancer Res 2011 31 9 2833 2839 21868526

[B48] Saydam O Senol O Schaaij-Visser TB Pham TV Piersma SR Stemmer-Rachamimov AO Wurdinger T Peerdeman SM Jimenez CR Comparative protein profiling reveals minichromosome maintenance (MCM) proteins as novel potential tumor markers for meningiomas J Proteome Res 2009 9 1 485 494 19877719 10.1021/pr900834h PMC2810358

[B49] Helfenstein A Frahm SO Krams M Drescher W Parwaresch R Hassenpflug J Minichromosome maintenance protein (MCM6) in low-grade chondrosarcoma: distinction from enchondroma and identification of progressive tumors Am J Clin Pathol 2004 122 6 912 918 15539383 10.1309/G638-TKNN-G2CJ-UXWL

[B50] Schrader C Janssen D Klapper W Siebmann JU Meusers P Brittinger G Kneba M Tiemann M Parwaresch R Minichromosome maintenance protein 6, a proliferation marker superior to Ki-67 and independent predictor of survival in patients with mantle cell lymphoma Br J Cancer 2005 93 8 939 945 16189522 10.1038/sj.bjc.6602795 PMC2361659

[B51] Wharton SB Chan KK Anderson JR Stoeber K Williams GH Replicative Mcm2 protein as a novel proliferation marker in oligodendrogliomas and its relationship to Ki67 labelling index, histological grade and prognosis Neuropathol Appl Neurobiol 2001 27 4 305 313 11532161 10.1046/j.0305-1846.2001.00333.x

[B52] Scott IS Morris LS Rushbrook SM Bird K Vowler SL Burnet NG Coleman N Immunohistochemical estimation of cell cycle entry and phase distribution in astrocytomas: applications in diagnostic neuropathology Neuropathol Appl Neurobiol 2005 31 5 455 466 16150117 10.1111/j.1365-2990.2005.00618.x

[B53] Soling A Sackewitz M Volkmar M Schaarschmidt D Jacob R Holzhausen HJ Rainov NG Minichromosome maintenance protein 3 elicits a cancer-restricted immune response in patients with brain malignancies and is a strong independent predictor of survival in patients with anaplastic astrocytoma Clin Cancer Res 2005 11 1 249 258 15671553

[B54] Facoetti A Ranza E Benericetti E Ceroni M Tedeschi F Nano R Minichromosome maintenance protein 7: a reliable tool for glioblastoma proliferation index Anticancer Res 2006 26 2A 1071 1075 16619508

[B55] Facoetti A Ranza E Grecchi I Benericetti E Ceroni M Morbini P Nano R Immunohistochemical evaluation of minichromosome maintenance protein 7 in astrocytoma grading Anticancer Res 2006 26 5A 3513 3516 17094475

[B56] Erkan EP Strobel T Lewandrowski G Tannous B Madlener S Czech T Saydam N Saydam O Depletion of minichromosome maintenance protein 7 inhibits glioblastoma multiforme tumor growth in vivo Oncogene 2013 doi:10.1038/onc.2013.423. [Epub ahead of print] 10.1038/onc.2013.423 24166506

[B57] Kobierzycki C Pula B Skiba M Jablonska K Latkowski K Zabel M Nowak-Markwitz E Spaczynski M Kedzia W Podhorska-Okolow M Dziegiel P Comparison of minichromosome maintenance proteins (MCM-3, MCM-7) and metallothioneins (MT-I/II, MT-III) expression in relation to clinicopathological data in ovarian cancer Anticancer Res 2013 33 12 5375 5383 24324072

[B58] Toschi L Bravo R Changes in cyclin/proliferating cell nuclear antigen distribution during DNA repair synthesis J Cell Biol 1988 107 5 1623 1628 2903166 10.1083/jcb.107.5.1623 PMC2115310

[B59] Ha SA Shin SM Namkoong H Lee H Cho GW Hur SY Kim TE Kim JW Cancer-associated expression of minichromosome maintenance 3 gene in several human cancers and its involvement in tumorigenesis Clin Cancer Res 2004 10 24 8386 8395 15623617 10.1158/1078-0432.CCR-04-1029

[B60] Parkins CS Darling JL Gill SS Revesz T Thomas DG Cell proliferation in serial biopsies through human malignant brain tumours: measurement using Ki67 antibody labelling Br J Neurosurg 1991 5 3 289 298 1892572 10.3109/02688699109005189

[B61] Torp SH Diagnostic and prognostic role of Ki67 immunostaining in human astrocytomas using four different antibodies Clin Neuropathol 2002 21 6 252 257 12489673

