# Correction to: Minichromosome Maintenance (MCM) family as potential diagnostic and prognostic tumor markers for human gliomas

**DOI:** 10.1186/s12885-019-5436-4

**Published:** 2019-03-22

**Authors:** Cong Hua, Gang Zhao, Yunqian Li, Li Bie

**Affiliations:** 1 Department of Neurosurgery of the First Clinical Hospital, Jilin University, 71 Xinmin St, Changchun, Jilin, 130021 China; 2 Department of Pathology and Laboratory Medicine, University of California, Irvine, USA

**Correction to: BMC Cancer**

**https://doi.org/10.1186/1471-2407-14-526**

Following publication of the original article [1], the author noticed that there are some errors with Table 1 and Table 2. Please see the correct tables below. The authors apologize for any inconvenience caused.

**Table 1:** Comparison of mRNA expression of MCMs in glioma tumor tissue by qRT-PCR and microarray analysis

Gene	Unique ID	Gene origin	Fold							*p* value					
			II/Normal	III/Normal	IV/Normal	III/II	IV/II	IV/III	(II-IV)/Normal	II → Normal	III → Normal	IV → Normal	III → II	IV → II	IV → III
MCM2	202107_s_at	qRT-PCR	1.97	3.98	4.52	2.02	2.29	1.14	3.5	1.59E-02	2.15E-04	3.97E-04	3.80E-03	6.10E-04	8.31E-02
		Microarray	1.68	3.74	5.21	2.23	3.10	1.39		3.42E-02	2.98E-05	1.75E-08	8.38E-09	7.03E-06	1.26E-06
MCM3	201555_at	qRT-PCR	1.51	3.35	4.29	2.22	2.84	1.28	3.1	1.37E-02	1.95E-03	2.73E-04	1.89E-02	1.10E-03	1.72E-03
		Microarray	1.84	2.62	2.91	1.42	1.58	1.11		4.85E-03	2.66E-05	3.71E-06	1.06E-02	9.77E-07	6.82E-04
MCM4	222036_s_at	qRT-PCR	2.07	4.29	6.68	2.07	3.23	1.56	4.3	6.21E-02	4.36E-02	2.53E-02	7.30E-02	4.26E-02	6.82E-02
		Microarray	1.93	2.47	3.93	1.28	2.04	1.59		7.19E-02	1.82E-02	3.68E-03	6.01E-03	2.89E-03	4.29E-03
MCM5	201755_at	qRT-PCR	1.77	3.51	6.05	1.98	3.42	1.72	3.8	1.49E-02	1.38E-03	5.05E-04	1.32E-02	1.67E-03	7.29E-03
		Microarray	0.95	1.63	2.89	1.72	3.04	1.77		5.91E-03	2.79E-03	5.81E-03	3.09E-04	8.59E-07	1.23E-03
MCM6	201930_at	qRT-PCR	1.55	3.89	4.95	2.51	3.19	1.27	3.5	3.96E-02	1.05E-02	1.16E-02	2.65E-02	1.17E-02	7.92E-02
		Microarray	2.65	2.88	4.75	1.09	1.79	1.65		9.12E-03	1.07E-04	6.77E-04	8.10E-09	5.24E-03	1.42E-03
MCM7	208795_s_at	qRT-PCR	1.53	3.08	4.52	2.01	2.95	1.47	3.0	3.82E-02	2.02E-03	9.81E-03	1.25E-02	1.20E-03	4.01E-03
		Microarray	1.31	2.82	3.61	2.15	2.76	1.28		1.59E-04	5.09E-04	3.80E-04	4.79E-08	9.28E-08	6.36E-02
MCM10	220651_s_at	qRT-PCR	2.17	3.59	5.05	1.65	2.33	1.41	3.6	3.90E-02	4.50E-04	1.46E-04	2.96E-02	1.88E-03	2.60E-03
		Microarray	6.92	23.87	27.96	3.45	4.04	1.17		1.37E-03	5.39E-08	7.61E-09	5.86E-07	2.59E-09	5.83E-02

MCM3 and MCM5, MCM7 and MCM10 were significant different between grade II, III and IV samples (*p* < 0.05). MCM2 and MCM6 expressions were significantly increased between grade II (low grade) and III-IV (high grade) samples (*p* < 0.05)

**Table 2:** Multivariate analysis on prognosis of the patients (*n* = 51)

Parameter	Risk ratio	95% CI	*p*
Sex	2.067	0.789–4.670	0.151
Age (≥40)	3.155	1.984–4.039	0.042
WHO (Grade 4)	3.0843	1.523–3.810	0.034
MCM2	8.519	6.280–10.508	0.004
MCM3	7.328	5.102–9.833	0.007
MCM4	0.506	0.843–1.441	0.477
MCM5	2.117	0.946–3.127	0.146
MCM6	2.873	1.377–4.073	0.090
MCM7	6.576	4.114–8.243	0.010
MCM10	0.029	0.015–1.093	0.937
