# Supplementary material for: Minichromosome Maintenance (MCM) Family as potential diagnostic and prognostic tumor markers for human gliomas
Source: BMC Cancer. 2014 Jul 21;14:526. doi: 10.1186/1471-2407-14-526 (PMC4223428; doi:10.1186/1471-2407-14-526)
Supplement: Additional file 1 — Primer sequences and amplification summary. [file 1471-2407-14-526-S1.doc]

Additional file 1 Primer sequences and amplification summary

| Gene | Primer Sequence [5′3′] | Amplification size (bp) | Amplification efficiency (%) |
| --- | --- | --- | --- |
| 18S rRNA | F: GGAGTATGGTTGCAAAGCTGA  R: ATCTGTCAATCCTGTCCGTGT | 129 | 96% |
| HPRT1 | F: TGAGGATTTGGAAAGGGTGT  R: GAGCACACAGAGGGCTACAA | 118 | 103% |
| MCM2 | F: GTGGATAAGGCTCGTCAGAT  R: GTCGTGGCTGAACTTGTT | 87 | 95% |
| MCM3 | F: GAGTGAATCCAGGTTGAAGG  R: GATTCTGTGAGGCGATTCAT | 96 | 99% |
| MCM4 | F: GGACAATCTGACATAGCAATT  R: AGTCACTGTCAGGAAATCA | 84 | 109% |
| MCM5 | F: GTTTGACAAGATGCGAGAA  R: CCTTGGCGATAGAGATGG | 77 | 110% |
| MCM6 | F: ACTGTTCCTGGACTTCTTGG  R: ACGAATCAGTTCCTCTGCTAAT | 82 | 91% |
| MCM7 | F: ACCGAGACAATGACCTAC  R: GCTATGTAACGCCTCATG | 115 | 98% |
| MCM10 | F: GCCAAGATTCTACATTGCTCAT  R: ATTGTCTTCCTCCTCATCCAT | 97 | 105% |
