# Supplementary material for: Minichromosome Maintenance (MCM) Family as potential diagnostic and prognostic tumor markers for human gliomas
Source: BMC Cancer. 2014 Jul 21;14:526. doi: 10.1186/1471-2407-14-526 (PMC4223428; doi:10.1186/1471-2407-14-526)
Supplement: Additional file 2 — The other most up-regulated 20 genes. [file 1471-2407-14-526-S2.doc]

Additional file 2 The other most up-regulated 20 genes

| Gene | Gene.Title | Chromosomal.Location | Unique_id | Regulate |
| --- | --- | --- | --- | --- |
| CDC20 | cell division cycle 20 homolog | chr1p34.1 | 202870_s_at | up |
| CENPF | centromere protein F, 350/400ka | chr1q32-q41 | 207828_s_at | up |
| ASPM | asp (abnormal spindle) homolog, microcephaly associated | chr1q31 | 219918_s_at | up |
| PARD3 | par-3 partitioning defective 3 homolog | chr10p11.22-p11.21 | 221527_s_at | down |
| KIF20A | kinesin family member 20A | chr5q31 | 218755_at | up |
| RRM2 | ribonucleotide reductase M2 polypeptide | chr2p25-p24 | 201890_at | up |
| NCAPG | non-SMC condensin I complex, subunit G | chr4p15.33 | 218662_s_at | up |
| SMYD2 | SET and MYND domain containing 2 | chr1q41 | 212922_s_at | up |
| KIAA0101 | KIAA0101 | chr15q22.31 | 202503_s_at | up |
| CCNB2 | cyclin B2 | chr15q22.2 | 202705_at | up |
| NUSAP1 | nucleolar and spindle associated protein 1 | chr15q15.1 | 218039_at | up |
| BIRC5 | baculoviral IAP repeat-containing 5 | chr17q25 | 202095_s_at | up |
| RAD51 | RAD51 homolog | chr15q15.1 | 205024_s_at | up |
| DLG7 | discs, large homolog 7 | chr14q22.3 | 203764_at | up |
| FN1 | fibronectin 1 | chr2q34 | 210495_x_at | up |
| SHOX2 | short stature homeobox 2 | chr3q25-q26.1 | 210135_s_at | up |
| TOP2A | topoisomerase (DNA) II alpha 170kDa | chr17q21-q22 | 201291_s_at | up |
| NDC80 | NDC80 homolog, kinetochore complex component | chr18p11.32 | 204162_at | up |
| TPX2 | TPX2, microtubule-associated, homolog | chr20q11.2 | 210052_s_at | up |
